# Integrated Internal Stabilization for Saddle Nose Surgery

**Published:** 2016-03

**Authors:** Ebrahim Karimi, Jalal Mehdizadeh, Shahin Bastaninejad, Mohammad Koohkan

**Affiliations:** 1 *Otorhinolaryngology Research Center, Amir’Alam Hospital, Tehran University of Medical Sciences, Tehran Iran.*

**Keywords:** Nasal reconstruction, Wired costal graft, Saddle nose deformity.

## Abstract

**Introduction:**

Correction of Saddle nose deformity is one of the most challenging issues in facial plastic surgery.

**Materials and Methods:**

In this study, a single structure in the form of L-strut was attempted to be created by using one 0.035" Kirschner wire and an autologous costal graft out of the 10th and 11th ribs. This study involved 13 cases, most of whom were traumatic. The corrective surgical techniques used in this study will be described in detail.

**Results:**

There was no warping, no rejection, and no infection in the created L-strut and patients’ satisfaction was very good during the follow up period.

**Conclusion:**

Surgical correction of a saddle-shaped nose using the described technique seems to be an acceptable and uncomplicated technique, and the cosmetic result is totally acceptable.

## Introduction

Correction of saddle nose deformity (SND) aims to improve both nasal breathing function and aesthetic aspects for the patient. It is amongst the most challenging issues in facial plastic surgery; therefore, several techniques using various graft materials have been proposed for nasal reconstruction (1-5).

Sustained improvement in respiratory function of the nose and aesthetic reconstruction of the nose with the lowest incidence of complications is the ideal aim of different treatment modalities. However, the method of choice which achieves all these goals is still not established. Graft infection, warping, resorption, and extrusion are the most important postoperative complications (6,7).

Among the most important concerns is infection and extrusion of the alloplastic materials. Studies report the risk of infection between 2-4% and the risk of implant extrusion between 0-9% (8,9).

In cases for which autogenous grafts were used, although infection was rarely reported, the risk of displacement and warping was still considered to be one of the complications in using these kind of grafts (10).

There is a popular trend which uses rib cartilage as the graft of choice in these patients. However, costal cartilage has a risk of warping after corrective operation. There are studies that focus on this issue, such as Kim et al., who tried to show that concentric carving of this cartilage will lead to less warping potentials (11).

In a study published by Professor Gunter in 1997 (2), internal stabilization of the graft with a Kirschner wire was used to reduce the complications of autogenous costal cartilage grafts. In this study, significant results for preventing warping of the graft was achieved through the use of a Kirschner wire. In their proposed method, Gunter et al used two pieces of costal cartilage and two separate pieces of a Kirschner wire.

In this paper, we attempt to introduce our preliminary report on a new method for correcting SND using stabilized costal cartilage with a single Kirschner wire, and the observed results in our series of 13 patients are also reported. Our aims for this kind of reconstruction were: creating a more stable framework, creating a cosmetically acceptable nasofacial angle, and obtaining less warping in saddle nose patient using rib grafts.

## Materials and Methods

*Subjects:*

This research has been approved by the Otorhinolaryngology research committee of Tehran University of Medical Sciences, and all subjects gave informed consent for the study.

In this study, 13 patients with saddle nose deformity ranging from moderate to severe deformity (grade 3 to 5-Tardy classification) (12), without underlying disease causing saddle nose deformity, underwent reconstructive rhinoplasty in 2009 to 2013 in Amir’Alam Hospital. Patients’ age was between 22 and 45 years (mean 32 years). Patients were followed for 26 months on average (5 to 57 months). Demographic data, degree, and cause of deformity in addition to the duration of follow-up are presented in (Table. 1).

**Table 1:** Demographic data, degree and cause of deformity and duration of follow-up period

**Patient** **number/** **gender** **/** **age**	**Degree** **of** **Deformity**	**Cause of** **Deformity**	**Duration ** **of Follow-up** ** (months)**
01/ Male/25	severe	trauma	24
02/ Male/22	severe	trauma	26
03/ Male/25	average	trauma	25
04/ Female/27	severe	trauma	31
05/ Female/34	average	previous rhinoplasty	16
06/ Male/24	severe	trauma	23
07/ Female/37	average	previous rhinoplasty	51
08/ Female/30	severe	trauma	52
09/ Male/22	severe	trauma	5
10/ Female/45	average	Idiopathic	20
11/ Female/28	average	trauma	57
12/ Female/33	severe	trauma	7
13/ Male/39	severe	trauma	12

*Surgical Technique* *:*

**Fig1:**
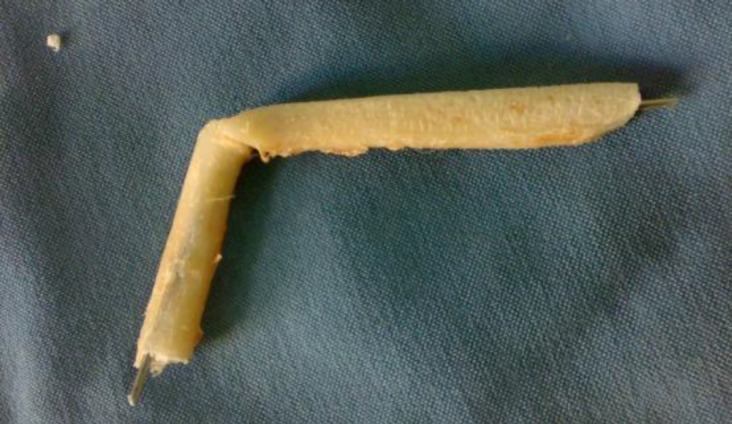
Integrated wired rib cartilages in a shape of L-strut

In this technique, after the 10^th^ and 11^th^ rib cartilages were harvested from the left side of the chest wall, with the 10^th^ cartilage being carved for the dorsum and the 11^th^ cartilage being carved for the columella. Then using a microdrill, the 0.035 inch Kirschner wire was passed through the core of both pieces of the prepared grafts. Then Kirschner wire was placed at approximately a 115 degree angle as an L-Strut. The Kirschner wire should have 5 to 8 mm of protrusions from both ends (Fig.1). 

When the graft is prepared, the rhinoplasty begins using an open approach. For graft replacement, one hole is drilled in the midline Nasion by microdrill and another hole is created in the pre-maxillary area, in lateral side of the anterior nasal spine on the edge of the upper rim of the pre-maxillary bone, using a fine drill. In order to prevent penetration of the palatal mucosa, a hole with a depth of 3-4 mm was drilled. The bony dorsum was corrected by rasping to create a predictable adhesion with the nasal dorsal graft during the post-operative period. The protruding ends of the Kirschner wire were inserted in the drilled holes and fixed to the medial crura of both sides in the columella area by suturing.

Tip plasty was performed and both upper lateral cartilages were sutured to the lateral aspect of the dorsal graft before surgery ended.

## Results

Of these patients, ten were traumatic cases, two patients were saddle nose due to the previous rhinoplasty operations, and one of case was idiopathic. The follow-up results for possible complications and patient satisfaction with their post-op noses (which was measured with Likert Scale) is shown in (Table.2).

**Table2:** Complications and the Degree of Patient Satisfaction

**Investigated** **Cases**	**Incidence**
Thoracic Surgical Site Infection	0 cases
Surgical area infection	0 cases
Nasal deviation of more than 5 ° from the sagittal plane	0 cases
graft resorption more than 50% in the profile view	0 cases
protrusion of kirschner wire	0 cases
Satisfaction(Likert scale-Maximum 5)	10 patients 5, 3 patients 4

**Fig 2:**
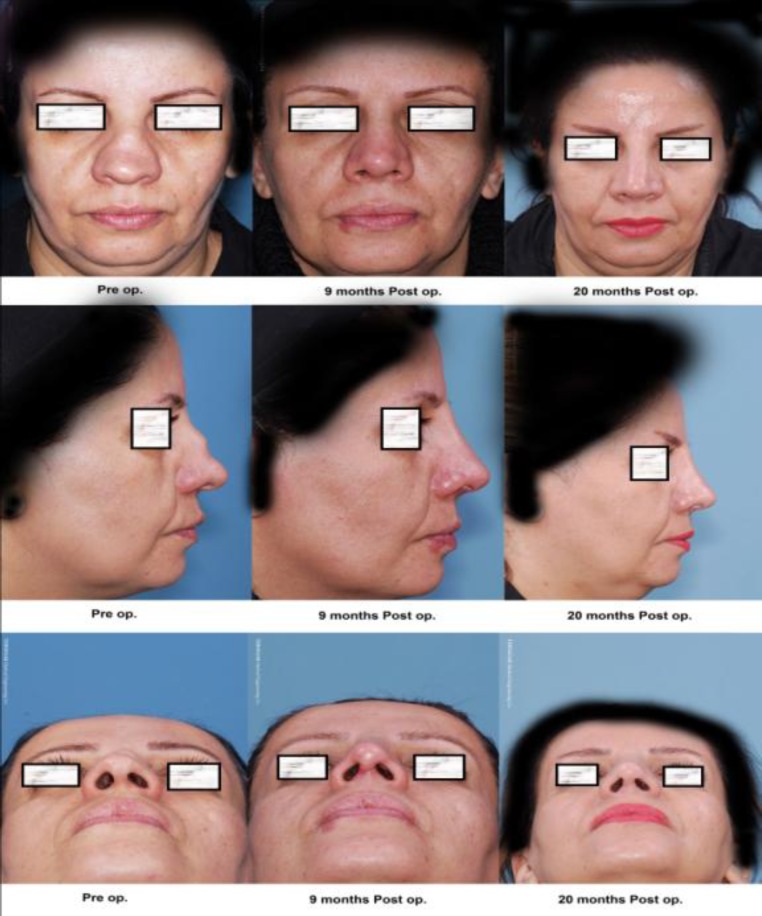
Post-up results for patient number 10

**Fig 3:**
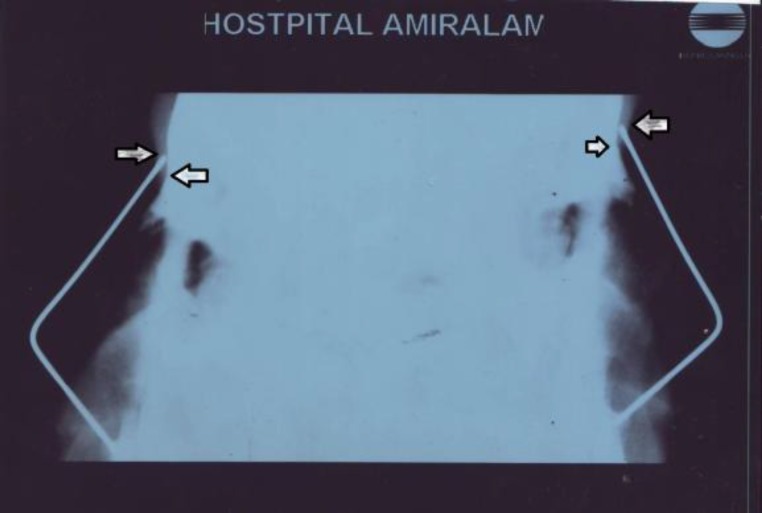
Lateral nasal X-ray of the same patient. The cephalic portion of the wire is

The post-up results for the 10^th^ patient are shown in Figure 2. In Figure 3 the lateral nasal graphy of the same patient is observed.

## Discussion

In the study done by Gunter (2), using a Kirschner wire effectively prevented postoperative graft warping, so that in contrast with the control group, with an average of 8.9° twists, in patients, for whom a Kirschner wire was used for reconstructing the nose, the warping was reduced to a mean 2.2°. In this study, a similar result was obtained. Additionally, it seems that the integrated internal stabilization of both grafts with one Kirschner wire will cause the frame work to be more reliable and more stable. In this study dorsum warping in patients is shown in Table 2 and was negligible in the sagittal plane.

This study has shown good results in terms of graft infection. For example, Park et al faced graft infection in 6% of their patients, who required antibiotic injections to control it (6). In the present study, no local or systemic signs of infection were observed. This was probably directly connected with establishing complete sterility during the extraction and preparation of the cartilage in addition to the loss of any underlying disease in the patients undergoing surgery.

Generally, none of the anticipated post-surgical complications occurred in our study. Perhaps this is due to the exclusion of the patients with saddle nose deformity caused by primary diseases such as Wegener's granulomatosis, leprosy, syphilis, and cocaine abuse.

## Conclusion

Based on this study, it seems that the use of autogenous costal cartilage reinforced with an integrated Kirschner wire is an appropriate option for patients with saddle nose deformity. However, this is a case series study and the results could not be generalized universally.
